# SARS-CoV-2: from its discovery to genome structure, transcription, and replication

**DOI:** 10.1186/s13578-021-00643-z

**Published:** 2021-07-19

**Authors:** Ayslan Castro Brant, Wei Tian, Vladimir Majerciak, Wei Yang, Zhi-Ming Zheng

**Affiliations:** 1 Tumor Virus RNA Biology Section, HIV DRP, National Cancer Institute, NIH, Frederick, MD USA; 2 Mechanism of DNA Repair, Replication, and Recombination Section, Laboratory of Molecular Biology, NIDDK, Bethesda, MD USA

## Abstract

SARS-CoV-2 is an extremely contagious respiratory virus causing adult atypical pneumonia COVID-19 with severe acute respiratory syndrome (SARS). SARS-CoV-2 has a single-stranded, positive-sense RNA (+RNA) genome of ~ 29.9 kb and exhibits significant genetic shift from different isolates. After entering the susceptible cells expressing both ACE2 and TMPRSS2, the SARS-CoV-2 genome directly functions as an mRNA to translate two polyproteins from the ORF1a and ORF1b region, which are cleaved by two viral proteases into sixteen non-structural proteins (nsp1-16) to initiate viral genome replication and transcription. The SARS-CoV-2 genome also encodes four structural (S, E, M and N) and up to six accessory (3a, 6, 7a, 7b, 8, and 9b) proteins, but their translation requires newly synthesized individual subgenomic RNAs (sgRNA) in the infected cells. Synthesis of the full-length viral genomic RNA (gRNA) and sgRNAs are conducted inside double-membrane vesicles (DMVs) by the viral replication and transcription complex (RTC), which comprises nsp7, nsp8, nsp9, nsp12, nsp13 and a short RNA primer. To produce sgRNAs, RTC starts RNA synthesis from the highly structured gRNA 3' end and switches template at various transcription regulatory sequence (TRS_B_) sites along the gRNA body probably mediated by a long-distance RNA–RNA interaction. The TRS motif in the gRNA 5' leader (TRS_L_) is responsible for the RNA–RNA interaction with the TRS_B_ upstream of each ORF and skipping of the viral genome in between them to produce individual sgRNAs. Abundance of individual sgRNAs and viral gRNA synthesized in the infected cells depend on the location and read-through efficiency of each TRS_B_. Although more studies are needed, the unprecedented COVID-19 pandemic has taught the world a painful lesson that is to invest and proactively prepare future emergence of other types of coronaviruses and any other possible biological horrors.

## Introduction

In early December 2019, an adult with atypical pneumonia of unknown etiology emerged in a central China city Wuhan, the capital of Hubei province. The disease had SARS-like characteristics of lymphopenia and bilateral ground-glass opacities in chest CT scans and was soon linked to the Huanan Seafood Market. However, the symptom onset date of the first identified patient who had no epidemiological link to the seafood market exposure was December 1, 2019, 33 days after the Wuhan 2019 Military World Game was carried out from October 18–27, 2019. The first 41 patients, with a cluster of family pneumonia cases, were admitted to hospitals by January 2, with six deaths by January 22 [1, 2]. However, the first confirmed case in Hubei of a resident aged 55 could be traced back to November 17, 2019 (South China Morning Post, March 13, 2020) or earlier to November 4 or even to mid-October as predicted by a coalescent framework modeling [3]. Deep sequencing analysis from lower respiratory tract samples indicated a novel coronavirus with > 75% sequence homology to SARS-CoV in the submitted clinical samples, which was named 2019 novel coronavirus (2019-nCoV). By January 5 of 2020, the whole genome sequence of 2019-nCoV was completed by Wuhan Institute of Virology, China CDC and Shanghai Public Health Clinical Center of Fudan University [4–6] and deposited immediately to the GenBank [5]. By January 7, 2020, a new coronavirus of probable bat origin using a host receptor ACE2 for human cell infection was isolated and characterized as an etiological agent of the 2019-nCoV [4, 7]. Subsequently, WHO named this mysterious pneumonia as coronavirus disease 2019 or COVID-19 and the ICTV named its etiological agent the SARS-CoV-2 [8, 9].

Wuhan, with a population of over 11 million people, was locked down on January 23, 2020 for quarantine to stop the arising respiratory tract transmission of COVID-19 from person to person. Rapid spread of COVID-19 to its neighboring cities, provinces and other countries in a short period of time caused a worldwide pandemic [1, 10]. By May 2, 2021, the Worldometer coronavirus (www.worldometers.info/coronavirus/) recorded more than 153.37 million COVID-19 infections, with 3.21 million deaths in 219 countries and territories. The United States alone had 33.1 million COVID-19 infections, with more than 590 thousand deaths. In the east coast state of Maryland, more than 380 thousand cases were confirmed with ~ 2% fatality by March 10, 2021, while in the West coast state of California at the same time, more than 3.5 million cases were reported with an overall fatality of 1.5% (Table 1). Although about one-third of COVID-19 deaths were age 70 and older in both USA states, the fatality rate of COVID-19 also varies among different ethnic groups, with the highest fatality of ~ 3.5% among the reported Asian cases (Table 1). The exact reasons for the higher SARS-CoV-2 fatality in Asian ethnic groups in the US remain to be investigated. Of note by April 1, 2021, the highest fatality rates of COVID-19 infections worldwide was 5.1% in China, 5.9% in Egypt and 9.0% in Mexico, when compared with an average of ~ 2.1% fatality rate among all other countries, including 1.8% in the US, 2.5% in Brazil, 3.3% in Peru, 2.0% in France, 2.2% in Russia, 2.9% in UK, 2.7% in Germany, 3.0% in Italy, 3.4% in South Africa, 3.3% in Iran, 1.7% in Saudi Arabia, 1.3% in India, 2.7% in Indonesia, 2.2% in Myanmar, 1.7% in Philippines, 1.9% in Japan, and surprisedly, only 0.3% in Thailand, 0.4% in Malaysia and no death in Laos. Moreover, fewer COVID-19 cases were reported in the latter three countries.

**Table 1:** COVID-19 infections and fatality rate among different age and ethnic groups in Maryland (A) and California (B) on March 10, 2021, date from https://coronavirus.maryland.gov/ and https://www.cdph.ca.gov/Programs/CID/DCDC/Pages/COVID-19/Race-Ethnicity.aspx sources

A: Maryland			
Age	Cases	Deaths	% Death
0–9	19,516	3	0.02
10–19	37,640	6	0.02
20–29	70,899	35	0.05
30–39	66,837	76	0.11
40–49	58,936	209	0.35
50–59	58,681	594	1.01
60–69	39,729	1240	3.12
70–79	22,513	1998	8.87
80+	14,815	3657	24.68
NA	0	2	
Total	389,566	7820	2.01

Race/ethnicity	Cases	Deaths	% Death
African-American	112,790	2684	2.38
Asian	9005	273	3.03
White	135,611	4014	2.96
Hispanic	62,511	717	1.15
Other	18,432	81	0.44
N/A	51,217	51	0.10
Total	389,566	7820	2.01

B: California			
Age	Cases	Deaths	% Death
0–17	452,443	14	0.003
18–34	1,175,329	744	0.063
35–49	839,387	2876	0.343
50–64	671,794	10,593	1.577
65–79	272,863	18,627	6.827
80+	102,873	21,727	21.12
Total	3,514,689	54,581	1.553

Race/ethnicity	Cases	Deaths	% Death
Latino	1,519,953	24,810	1.63
White	550,982	16,834	3.06
Asian	188,068	6246	3.32
African- American	112,115	3329	2.97
Multi-Race	43,554	712	1.63
American Indian or Alaska Native	9183	188	2.05
Native Hawaiian and other Pacific Islander	15,407	324	2.10
Other	304,006	1122	0.37
NA	773,594	1025	0.13
Total	3,516,862	54,590	1.552

## Zoonotic *Coronaviruses* and the possible origin and transmission of SARS-CoV-2

SARS-CoV-2 belongs to the beta-coronavirus genus of the family *Coronaviridae*, which consists of 4 genera: *alpha-coronavirus*, *beta-coronavirus*, *gamma-coronavirus*, and *delta-coronavirus* (ICTV Virus Taxonomy: 2019 Release). Coronaviruses are enveloped viruses with a single-stranded, positive-sense RNA genome of 29–30 kb in size and infect numerous animal species including humans [11]. Many exhibit high interspecies transfers and thus are important zoonotic pathogens. Bats and birds are considered the “natural reservoirs” for human coronavirus zoonotic infections. As of today, there are seven human coronaviruses (hCoV), including two alpha-coronaviruses hCoV-229E and hCoV-NL63 and five beta-coronaviruses hCoV-HKU1, hCoV-OC43, SARS-CoV, MERS-CoV, and SARS-CoV-2 (Table 2). Patients infected by hCoV-229E, hCoV-NL63, hCoV-OC43, and hCoV-HKU1 manifest only common cold [12]. However, SARS-CoV, MERS-CoV and SARS-CoV-2 cause severe acute respiratory syndrome (SARS). SARS-CoV was first recognized as the etiological agent of the SARS outbreak of 8437 cases with a high fatality rate of ~ 10% in winter 2002, initially in Guangdong province in Southern China and later in more than 30 countries [13, 14]. Middle Eastern respiratory syndrome (MERS) with a fatality rate of ~ 34% was caused by MERS-CoV in 2012 first in Saudi Arabia and then spread to 27 countries with total of ~ 2500 cases [15, 16].

**Table 2:** Human coronaviruses

Genera	Strain	Discovery	Receptor	Symptoms
Alpha-coronavirus	hCoV-229E	1966	Aminopeptidase N (CD13)	Mild
	hCoV-NL63	2004	ACE2	Mild
Beta-coronavirus	hCoV-OC43	1967	9-*O*-Acetylate sialic acid	Mild
	hCoV-HKU1	2005	9-*O*-Acetylate sialic acid	Mild
	SARS-CoV	2003	ACE2	Severe
	MERS-CoV	2012	DPP4	Severe
	SARS-CoV-2	2020	ACE2	Severe

*ACE2* angiotensin 1 converting enzyme 2, *DPP4* dipeptidyl peptidase 4

All human coronaviruses are believed to be a result of the zoonotic transfer (“spillover”) from animal reservoirs either directly or through an intermediate animal host [17, 18]. Though hCoV-OC43 and hCoV-HKU1 are probably originated from rodents [19], bats are the reservoir of most coronaviruses, which are spilled over to humans probably through an intermediate host, such as civets (SARS-CoV) [20, 21] or camels (MERS-CoV) [22, 23]. SARS-CoV-2 with possible bat origin via an unknow intermediate host was proposed because its genome sequence is 96.2% identical to a bat coronavirus RaTG13 from Yunnan province of Southern China [4]. This hypothesis had been carefully discussed [24] and was further supported by another finding that one of four SARS-CoV-2-like bat coronavirus genomes, RpYN06, from Yunnan province exhibits 94.5% sequence identity to the SARS-CoV-2 genome. The other three are identical in sequence to a pangolin SARS-CoV-2-like coronavirus identified in the neighboring Guangxi province [25]. Moreover, human-to-animal transmission of SARS-CoV-2 has been reported for dogs, cats, lions, tigers, and minks [26–29]. More strikingly, transmission of the SARS-CoV-2 D614G strain from humans to minks and back to humans was evident in mink farms in Southeastern Netherlands [29, 30].

## SARS-CoV-2 genome structure and expression

Like other hCoVs, SARS-CoV-2 has a single-stranded, positive-sense RNA (+RNA) genome of 29,882 [31], 29,891 [4] or 29,903 nucleotides (nts) [5]. The genome is packed by viral nucleocapsid (N) proteins as a large ribonucleoprotein (RNP) complex and enclosed by an envelope membrane with lipids and viral proteins S (surface or spike), M (membrane) and E (envelope). The SARS‐CoV‐2 genome exhibits significant genetic diversity since its discovery (https://nextstrain.org/sars-cov-2) and has displayed over 7123 unique single nucleotide mutations/modifications among 12,754 complete US genome sequences by September 11, 2020 [32], or 29% of the genome positions over forty thousand SARS-CoV-2 genomes worldwide [33]. Host RNA editing machinery, of which ADAR deaminases target dsRNA for deamination of adenosines into inosines (A-to-I) and APOBECs deaminate cytosines into uracils (C-to-U) on ssRNA or ssDNA, may contribute to the observed SARS-CoV-2 genome mutations/modifications during virus infection [34, 35]. The SARS-CoV-2 genome is unstable at elevated temperature because of highly enriched A+U content (62%) and reduced G+C content (38%), which is comparable to the hCoV-OC43 genome (63% A+U and 37% G+C) and the hCoV-NL63 genome (66% A+U and 34% G+C). The SARS-CoV-2 genome, like all other hCoVs, such as SARS-CoV and MERS-CoV, has a m^7^G-cap structure, m^7^GpppA_1_, on the genome 5′ end [36] and a ~ 30–60-nt-long (47 nts in median length) poly-A tail on its 3′ end for viral genome stability and preventing cellular exoribonuclease digestion [35]. The 5′ untranslated region (UTR) of the SARS-CoV-2 genome is 265-nt long, longer than hCoV-OC43 (209 nts), but shorter than hCoV-NL63 (286 nts). It contains a 72-nt-long 5′-leader, a transcription regulatory core sequence (TRS_L_, ACGAAC), and several other cis-elements to regulate viral translation, subgenome synthesis and viral genome packaging [37, 38], and to confer resistance to degradation of viral mRNAs. Secondary structure prediction of the SARS-CoV-2 5′ UTR indicates the presence of five stem-loops [39] and a very stable four-way junction close to the AUG start codon of ORF1a [37].

The 3′ UTR of the SARS-CoV-2 genome is 337-nt long, longer than both hCoV-OC43 (286 nts) and hCoV-NL63 (287 nts), but shorter than the other two non-hCoVs, mouse hepatitis virus (MHV, 436 nts) and pig transmissible gastroenteritis virus (TGEV, 492 nts). The viral 3′ UTR contains the binding site of the replication and transcription complex (RTC) important for initiating replication and transcription of the intermediate negative-sense RNA (−RNA). The presence of cis-acting elements, such as a bulged stem-loop (BSL) and a pseudoknot, at the 3′ UTR in a model beta-coronavirus MHV and alpha-coronaviruses hCoV-229E and hCoV-NL63, were reported to be essential for binding of the MHV RdRP and viral genome transcription and replication [40, 41]. The SARS-CoV-2 3′ UTR also contains an octanucleotide sequence 5′-GGAAGAGC-3′ with unknown function at the location of ~ 70–80 nts from the 3′-end of the viral genome across all genera of the *Coronaviridae*, and a non-essential hyper-variable region (HVR) [39, 41, 42]. Like other coronaviruses, the 3′ UTR of SARS-CoV-2 has no canonical polyadenylation signal sequence AAUAAA. Thus, polyadenylation of viral RNAs is most likely carried out by a viral adenylyltransferase nsp8 [43].

**Fig. 1:**
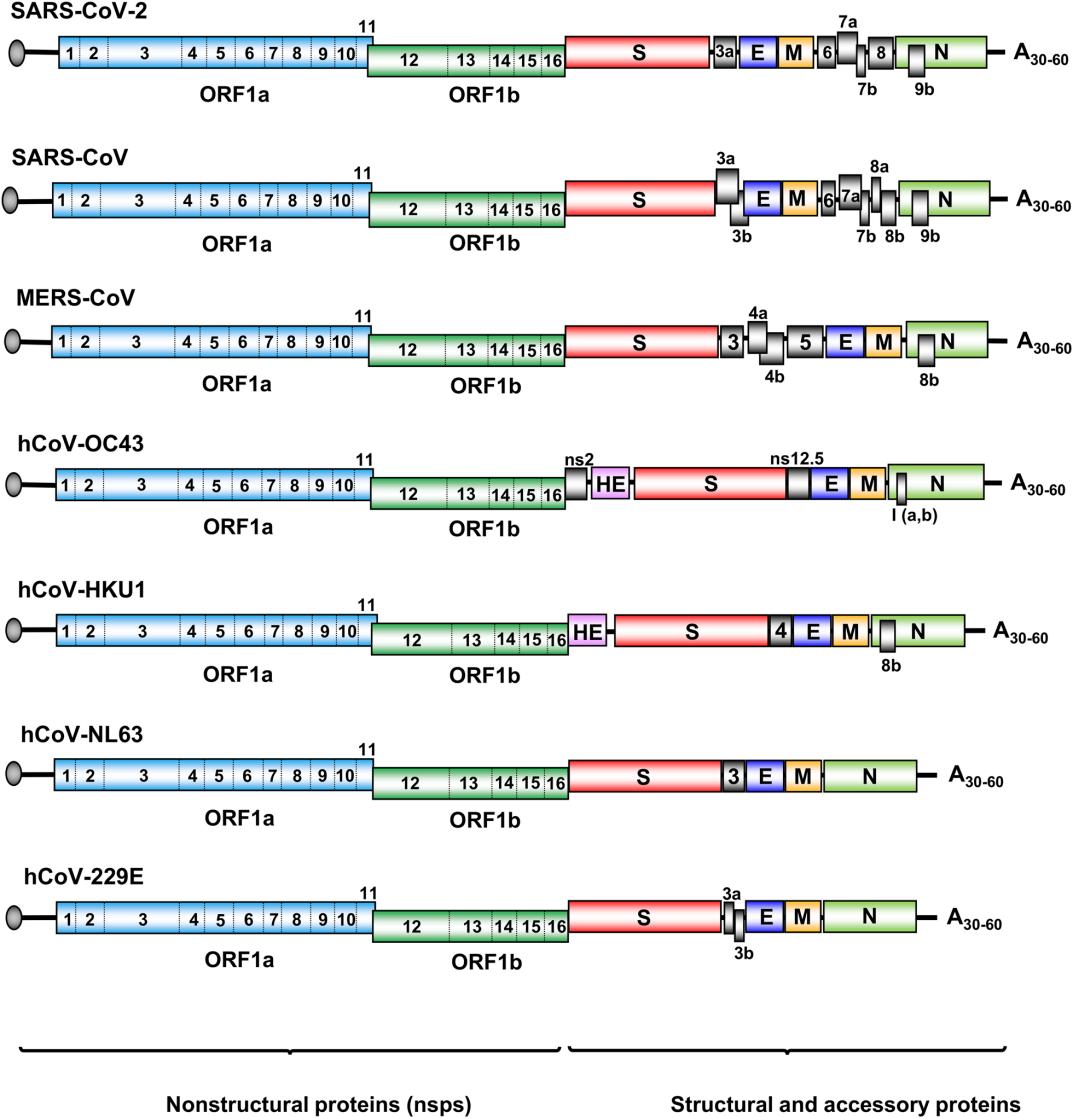
Genome structure and coding potentials of human coronaviruses. The viral genome is a single-stranded, positive-sense RNA with a cap (grey circle) at the 5′-end and a poly-A tail (A30-60) at the 3′ end. The genome encodes 16 non-structural proteins (ORF1a → nsp1-11 and ORF1b → nsp12-16) from the left three-fourth of the genome, and 4–5 structural proteins (S, spike; E, envelope; M, membrane; N, nucleocapsid; HE, hemagglutinin esterase) and various number of accessory proteins (numbered boxes) from the right one-fourth of the genome

Although different from SARS-CoV and other hCoVs in numbers of encoded accessory proteins and lacking a hemagglutinin esterase (HE) gene found in hCoV-OC43 and hCoV-HKU1 (Fig. 1), the SARS-CoV-2 genome has the coding capacity and strategies for nonstructural proteins (nsps) and structural proteins, which resembles all other coronaviruses (Fig. 1). The SARS-CoV-2 genome encodes 16 nonstructural, 4 structural, and 6 accessory proteins (Fig. 1). All 16 nsps involving in viral RNA transcription, replication and immune evasion are cleavage products of two polyproteins encoded by the ORF1a and ORF1b, which together occupy approximately 70% of the viral genome from the 5′ end. Structural proteins S, E, M and N for virion formation and the accessory proteins (3a, 6, 7a, 7b, 8, and 9b) with unknown function are encoded together by the rest of 30% viral genome on the 3′ end (Fig. 1). Although ORF3b (22 aa residues) [44] and ORF3c (41 aa residues) [45] overlapping SARS-CoV-2 ORF3a were predicted and ectopic ORF3b showed anti-IFN activities [44], their authentic expression and activities in SARS-CoV-2 infection remain to be verified. Additional upstream and internal ORFs, including ORF10, might exist in the SARS-CoV-2 genome based on computer prediction [35, 37, 39, 46] and ribosome profiling [47], but require further laboratory validation.

As the largest RNA genome among all RNA viruses, the positive-sense genome of SARS-CoV-2 directly translates two polyproteins from the ORF1a and ORF1b in the cytoplasm as soon as the virus gets into a susceptible cell. Because ORF1a and ORF1b partially overlap and ORF1b is in the − 1 reading frame relative to ORF1a, expression of ORF1b requires a programed − 1 ribosomal frameshift, for which the mechanism is not fully understood [48]. Cleavage of the two polyproteins by two self-activating viral proteases (Papain-like protease PLpro or nsp3 and 3-chymotrypsin-like protease 3CLpro or nsp5) produces 16 nsps. However, all other viral structural proteins and accessory proteins have to be translated from newly synthesized viral subgenomic RNAs (sgRNA) containing a 72-nt-long 5′ leader derived from the viral genome 5′-end. A search for Kozak sequence with each AUG initiation codon of individual ORFs for efficient translation [49] shows a required purine A or G at the − 4 position in ORF1a, S, M, 7a and 7b, 8 and N and a G at the + 4 position in ORF1a, 3a and M [50]. Thus, not every ORF in the SARS-CoV-2 genome has a Kozak sequence. How SARS-CoV-2 utilizes host translational machineries for viral protein production, in particular for those ORFs without the Kozak sequence, remains largely unexplored. Like other coronaviruses, SARS-CoV-2 genome does not contain any known internal ribosomal entry sequence (IRES) [50].

Among 16 nsps from the smallest nsp11 (13 aa residues) to the largest nsp3 (1299 aa residues) [51], some of their functions have been determined and summarized as follows [52, 53]. Nsp1 occupies the ribosomal mRNA-binding channel to inhibit translation of host proteins [54]; nsp2 binds host prohibitin 1 and 2 and may play a role in disrupting the host cell environment [51]; nsp3 is a papain-like protease for viral polyprotein processing; nsp4 and nsp6 form double membrane vesicles (DMVs) associated with replication–transcription complexes; nsp5 is a 3C-like protease for viral polyprotein processing; nsp7 and nsp8 are accessory factors of RdRP; nsp8 functions as a primase and also an RNA 3′-terminal adenylyltransferase (TATase) activity [43]; nsp9 is a RNA-binding protein [55, 56]; nsp10 is a cofactor of nsp14 and nsp16; nsp11 is an intrinsically disordered protein with unknown function [57]; nsp12 is an RNA-dependent RNA polymerase (RdRP) [58] and also a nucleotidyltransferase; nsp13 is a helicase; nsp14 is a proofreading 3′–5′ exoribonuclease and a guanosine-N7 methyltransferase (N7-MTase) for the RNA cap formation; nsp15 is a uridine-specific endoribonuclease and interferon antagonist; nsp16 is a ribose 2′-*O*-methyltransferase for genomic RNA cap formation.

Among viral structural and accessory proteins, which are expressed only from newly synthesized individual sgRNAs, the S, M and E proteins are incorporated into viral envelope (membrane) for virion formation. The trimeric S protein on viral envelope specifically binds to a cellular receptor, angiotensin-converting enzyme 2 (ACE2), for viral entry into susceptible cells, and thus initiates the first step of virus infection [4, 59–61]. Host cell transmembrane serine protease 2 (TMPRSS2) serves as a S protein activating protease [62, 63]. The E protein creates an ion channel in the viral membrane and probably plays a role in pathogenicity [64, 65]. The N protein binds the viral genomic RNA (gRNA) and packs the gRNA as a ribonucleoprotein complex in the virions [66]. The M protein is a transmembrane glycoprotein important for viral morphogenesis and budding by interacting with S, E and N proteins [67]. The number of accessory proteins encoded by different coronaviruses (Fig. 1) remains under debate as their coding potentials are based primarily on bioinformatic prediction [68]. Functions of all accessory proteins are poorly understood and might regulate host immunity and viral adaptation [69, 70].

## SARS-CoV-2 genome replication and transcription

**Fig. 2:**
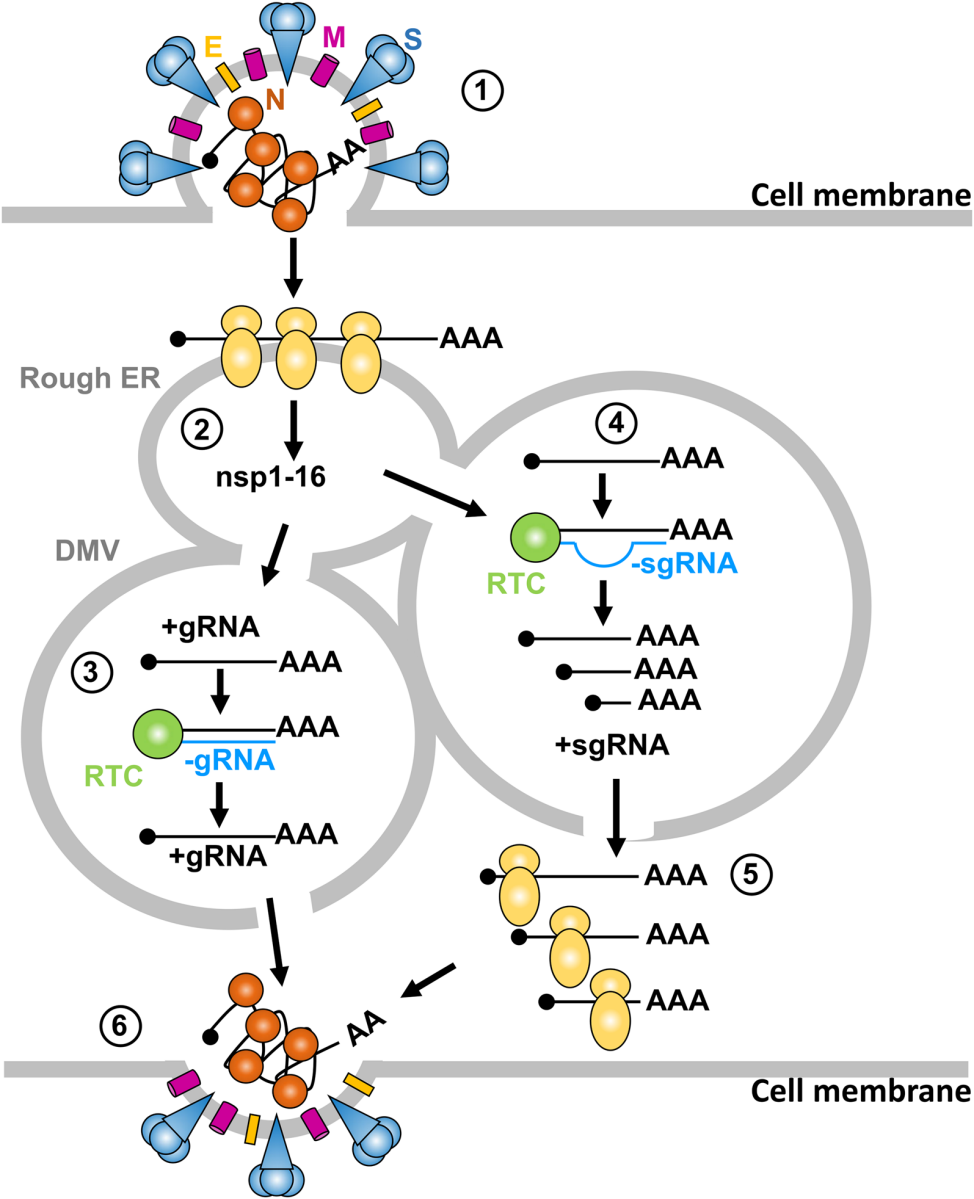
Coronavirus genome replication and transcription. Diagram showing the key steps in coronavirus entry (1), initial translation of incoming viral +gRNA to express viral non-structural proteins (nsp1-16) (2), genome replication in double-membrane vesicles (DMVs), continuous transcription of gRNA through a −gRNA-intermediate by viral replication and transcription complex (RTC) (3), generation of sgRNA by discontinuous transcription RTC (4), the expression of structural and accessory proteins from +sgRNA (S, spike; M, membrane; E, envelope; N proteins) (5), and virion assembly and release (6)

Similar to SARS-CoV, SARS-CoV-2 infection starts with virion attachment to the target cells mainly via interactions of the S proteins with host-cell receptor ACE2 [4, 59–61]. Proteolytic cleavage of the S protein by TMPRSS2 results in structural changes of the S protein that initiates the fusion of viral and host membrane and release of the viral gRNA into the cytoplasm (Fig. 2 step 1). Both ACE2 and TMPRSS2 are expressed in many cell types, with particularly high expression in lungs and intestine epithelia and endothelial cells, allowing SARS-CoV-2 to target numerous vital organs [62, 71–73]. As an RNA virus, SARS-CoV-2 replicates exclusively in the cytoplasm of infected cells, where the viral genome is first unpacked from bound viral N proteins by cellular proteases. The viral +gRNA then serves directly as an mRNA for translation of the ORF1a and ORF1b (Fig. 2 step 2) and also as a template RNA for −RNA transcription (Fig. 2 steps 3 and 4). Subsequent interactions of the nsps including viral RdRP, derived from cleaved ORF1a and ORF1b polyproteins, lead to formation of a replication and transcription complex (RTC) on the template +gRNA for virus gRNA transcription (Fig. 2 step 3) and sgRNA synthesis (Fig. 2 step 4) inside virus infection-induced DMVs [74, 75]. The newly synthesize sgRNAs released from the DMV encode viral structural and accessory proteins (Fig. 2 step 5). Finally, a newly generated gRNA is encapsidated with N proteins, enclosed by a viral envelope and released from the infected cells [66] (Fig. 2 step 6). The mystery in the final step is why only one of the newly synthesized viral full-length +gRNAs is packed into each virion, and how the +gRNAs are distinguished from +sgRNAs during SARS-CoV-2 virion assembly?

**Fig. 3:**
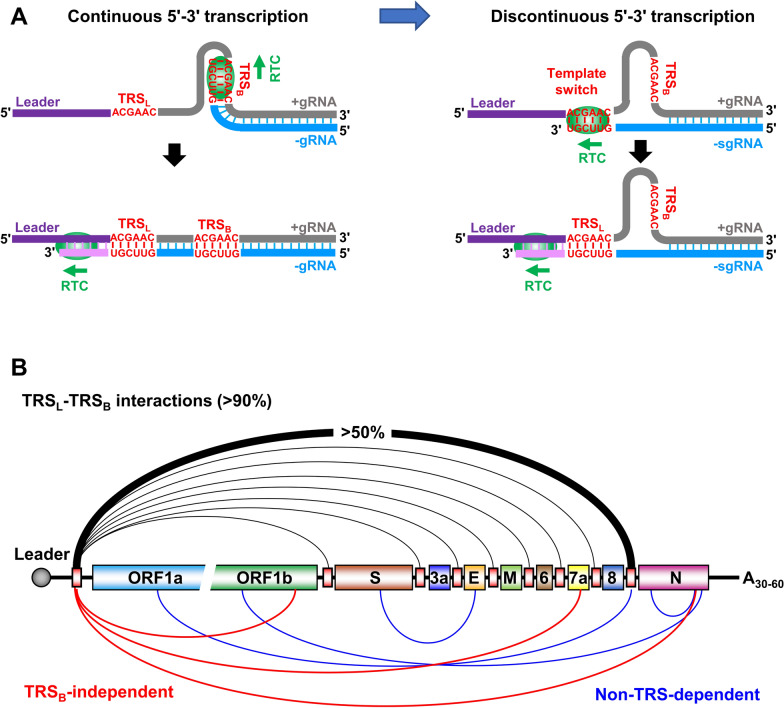
A proposed model of viral RNA transcription and template switch during SARS-CoV-2 infection. **A** Continuous 5′–3′ transcription of viral genomic +gRNA leads to synthesis of the full-length, negative-sense viral genomic RNA (−gRNA) (left). Because RTC-mediated RNA transcription starts from the highly structured viral gRNA 3′ end, this transcription often leads to discontinuous 5′–3′ transcription by proposed template switch (right). Through interactions between transcription regulatory sequences (TRS) located in the leader (TRS_L_) and the genome body (TRS_B_), the template switch results in the production of viral subgenomic RNAs (−sgRNAs). **B** Diagram of SARS-CoV-2 genome with predicted ORFs (colored boxes) and TRS (smaller red boxes) upstream of individual ORFs. Above are the canonical TRS_L_-dependent junctions detected in the individual sgRNAs from SARS-CoV-2-infected cells by RNA-seq, with the junction reads corresponding to the sgRNA encoding N protein being the most abundant. Below are the TRS_B_-independent interactions of TRS_L_ (red) and non-TRS dependent (blue) junctions detected by RNA-seq with unknown function [35, 38]

How SARS-CoV-2 induces DMV biogenesis remains to be elucidated and may require virus-induced invaginations of cellular membranes and excessive membrane-remodeling [75]. Viral transcription is presumably confined in DMVs with concentrated viral nsps and host factors. The newly formed RTCs inside DMVs synthesize viral +gRNA and numerous +sgRNAs efficiently via an intermediate negative-sense −gRNA. The DMVs provide physical separation of these RNAs from the immune sensors in the cytoplasm to evade host innate immunity. Although not fully understood, emerging evidences indicate that SARS-CoV-2 transcription resembles other coronaviruses [76]. After RTC formation in DMVs, RTC binds to the +gRNA 3' end to initiate the continuous transcription of a full-length, −gRNA intermediate (Fig. 3A, left). This −gRNA can be then used as a template by RTC to transcribe viral positive-sense +gRNAs. However, RTC transcription of +gRNA also leads to discontinued transcription, thus producing −sgRNAs [76]. The mechanism of producing −sgRNAs is likely that the RTC pauses on specific sites containing the transcription regulatory sequence (TRS, ACGAAC in both SARS-CoV and SARS-CoV-2) [38, 77] to synthesize −sgRNAs through interacting with a viral 5' leader by template switch skipping (deleting) the internal RNA regions (Fig. 3A, right).

The molecular mechanism of this discontinuous synthesis remains to be investigated. Viral RNA-seq analyses from SARS-CoV-2 infected cells support such a template switch presumably through long-range base-pairing between distal elements [35, 38, 78] (Fig. 3B). In this proposed template switch or jumping model, the RTC complex might temporarily dissociate from the 3' half of +gRNA template to grasp the 5'-end leader, leading to skipping a large part of the internal genome (Fig. 3A, right). This is mediated presumably by the interaction of a TRS within the 5' end leader (TRS_L_, ACGAAC) with the TRS in the viral genome body (TRS_B_) upstream of each individual structural/accessory gene (Fig. 3B). Through the sequence complementarity between TRS_L_ and TRS_B_, of which variations in its 6–7 core sequence are often seen in different coronaviruses, this RNA–RNA interaction-mediated template switch results in discontinuous transcription of SARS-CoV-2 genome and a collection of individual −sgRNAs with variable sizes [38, 78]. These −sgRNAs could be then used as templates to synthesize individual +sgRNAs [77, 79, 80]. Conceivably, this model might lead to bidirectional template switches for both −sgRNA and +sgRNA synthesis in the cells infected by SARS-CoV and SARS-CoV-2 [38, 77]. Consequently, all +sgRNAs in different sizes have the same +gRNA 5' leader sequence and the same 3' half of the viral genome. Typically, each +sgRNA translates one protein from the first ORF within the +sgRNAs. The intermediate −gRNAs and −sgRNAs are less abundant in the infected cells and functionally might not code any viral proteins. Although the majority (90%) of sgRNAs are disproportionately generated by a leader-dependent template switch between TRS_B_ and TRS_L_, a small fraction (< 10%) of sgRNAs might be produced in a TRS_B_-independent or even in a non-TRS-dependent way (Fig. 3B) [35, 38, 78], indicating that aberrant RNA–RNA interactions induced by certain RNA structures or binding of viral and cellular factors can occur in these template switch events. Findings of the multiple site interactions between host small nuclear RNAs (U1, U2 and U4 snRNAs) and virus RNAs suggest high complexities of RNA–RNA interactions in the infected cells [78].

While the presence of a 5'-end cap was confirmed on both +gRNA and +sgRNA species, it is unknown whether the viral −gRNA and −sgRNA intermediates are also capped during SARS-CoV-2 transcription and post-transcriptional RNA processing. The lack of a cap on −gRNA and −sgRNA would render the newly synthesized viral −RNA unstable and explain their low abundance in infected cells. As a cytoplasmic RNA virus, the cap structure cannot be added to viral RNAs by the host nuclear capping machinery. Instead, the viral RNA capping in all coronaviruses, including SARS-CoV and SARS-CoV-2, is carried out by the following four viral proteins, several of which are bifunctional. nsp10 activates nsp14 and nsp16 [81, 82]; nsp13 is both an RNA helicase and RNA/NTP triphosphatase (helicase/RTPase) [83]; nsp14 is a 3'–5' exonuclease that removes mismatches and mRNA cap guanine-N7 methyltransferase (N7-MTase) [81, 84]; nsp16 is a cap ribose 2'-O methyltransferase (2'-O-MTase) and a guanylyl transferase [85]. The first step for the RNA capping is the hydrolysis of the ppp-RNA by the RTPase activity of nsp13 to generate a 5' pp-RNA [83]. Subsequently, the pp-RNA receives a GMP moiety becoming a Gppp-RNA, which is methylated efficiently at the N7 site by the N7-MTase of the nsp14 in complex with nsp10 [81, 86, 87]. Lastly, the 2'-O-MTase activity of nsp16, activated by the cofactor nsp10, converts the viral RNA from an uncapped (cap-0) to capped form (cap-1) by transferring a methyl group to the first nucleotide, usually adenosine, on the ribose 2'-O position of the viral RNA [88], finalizing the capping. This has been supported by direct observation of nsp16-nsp10 heterodimer formation at the 5' end of SARS-CoV-2 RNA and addition of a methyl group to the first nucleotide of the 5' end of viral mRNA [36, 82]. The efficiency of this capping process remains to be investigated. Whether there is any control steps to ensure that only capped viral RNAs leave the DMVs is unknown.

There is almost no report of SARS-CoV-2 RNA polyadenylation up to date. The newly synthesized SARS-CoV-2 +gRNA has a ~ 30–60-nt-long (47 nts in median length) poly-A tail on its 3' end [35]. Since hCoV RNA genomes don’t have a conventional poly-A signal and are transcribed in the cytoplasm in the infected cells, the polyadenylation found in hCoV-229E RNAs is likely carried out by a viral adenylyltransferase nsp8, which can be stimulated by a short U-stretch in the RNA template in the presence of divalent metal ions Mg^2+^ or Mn^2+^ [43]. Such U-stretch sequences exist in all isolated SARS-CoV-2 genomes. It has been shown that the poly-A tail length is correlated with the infection stage in other coronaviruses, reaching to ~ 60 nts in the early stage of infection and gradually reducing to ~ 30 nts in the later stage [89, 90]. The mechanism of how coronaviruses regulate the poly-A tail length remains unknown. A longer CoV-poly-A tail facilitates better translation efficiency [89] and may play a role in preventing RNA turnover better [91]. It has been reported that an AGUAAA hexamer motif could be an important cis-element in bovine coronavirus polyadenylation of the nascent RNA [92]. The SARS-CoV-2 genome 3' end contains a motif AAGAA, which is subjected to RNA modification (m6A, 5mC, and deamination, etc.) [35]. The modified RNAs were found to carry shorter poly-A tails than unmodified RNAs, suggesting a link between the internal modification and 3′ end tailing [35]. Whether the viral −gRNAs and −sgRNAs have a poly-A tail or whether the +gRNA and +sgRNA have a different length of the poly-A tails are untouched topics in the coronavirus field.

## Structures of RTC and RTC inhibitors

The virus-encoded RTC complex carries out all RNA synthesis. The core of RTC consists of RdRP (nsp12) and three accessory subunits: one nsp7 and two copies of nsp8 [93]. Copying RNAs full of secondary and tertiary structures is likely facilitated by nsp13, the ATP-dependent 5′ to 3′ RNA helicase. Nsp9/10/14 and nsp16 have been shown to regulate the RNA 5′ cap synthesis and stabilize genomic RNAs.

**Fig. 4:**
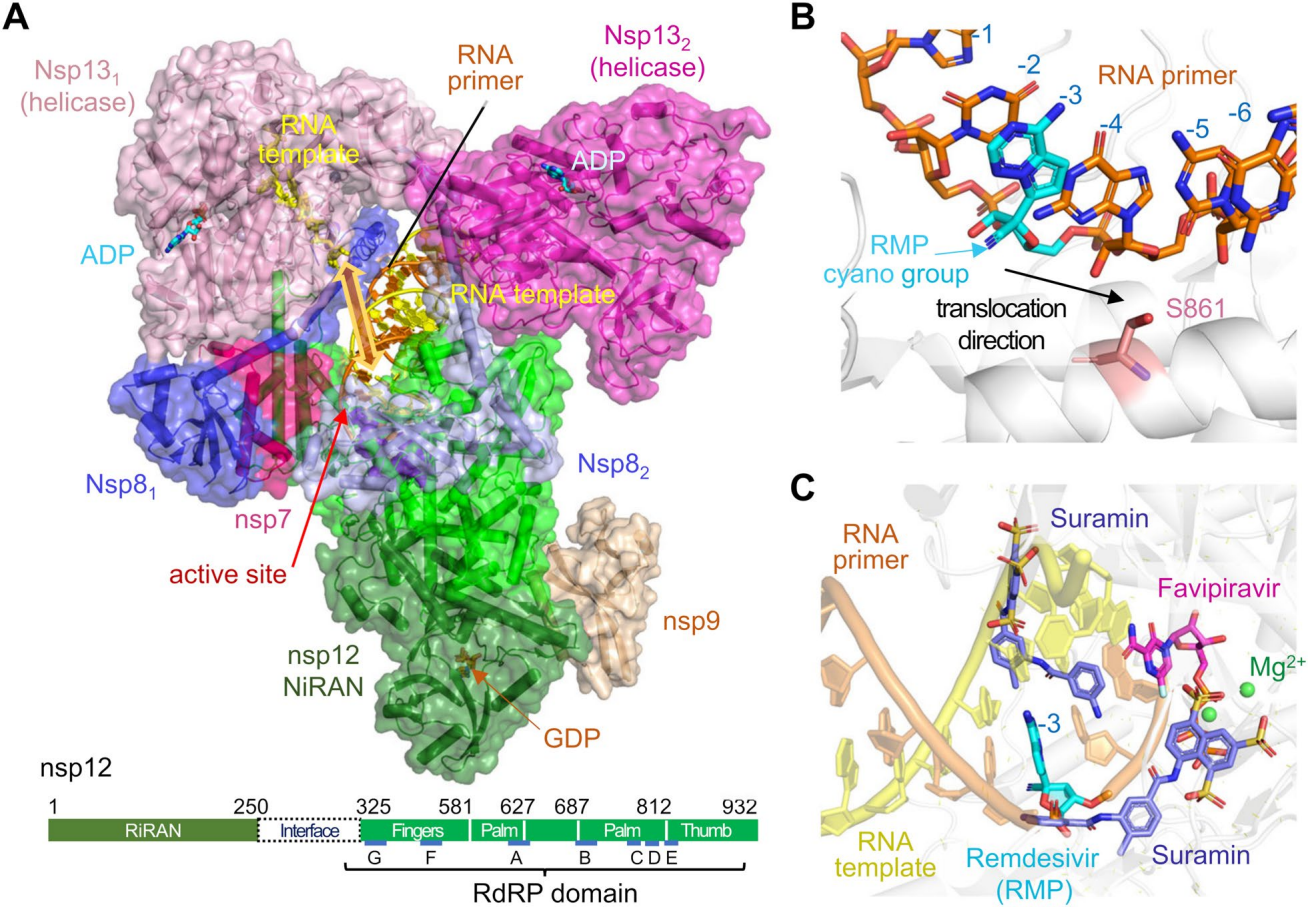
Structures of SARS-CoV-2 replication and transcription complex (RTC). **A** A composite structure of RTC from three PDB coordinates, 7CXM (architecture of nsp7, nsp8 X2, nsp12 and nsp13 X2 bound to RNA template and primer), 6XEZ (the ADP·AlF3, bound in the nsp13 helicase active site), and 7CYQ (nsp9 associated with nsp12 and GDP in the active site of RNA capping). The RNA template pieces bound to nsp13 and nsp12 are not connected and would be pulled by the two enzymes in opposite directions as indicated by the yellow double arrowheads. **B**, **C** Zoom-in views of RTC bound to inhibitors, Favipiravir (PDB: 7AAP), Remedisivir (RMP) (PDB: 7B3B), and Suramin (PDB: 7D4F). RdRP (nsp12) is shown in grey in **B**, **C**, the three inhibitors are in distinct colors. With several SO_4_ groups mimicking the phosphate backbone of RNA, two Sumarin molecules (cyan) compete for the RNA template and primer binding. Remedisivir (blue) is already incorporated in the RNA primer strand at − 3 position. Favipiravir RTD (magenta) occupies the incoming nucleotide position, but the phosphates are in a non-productive conformation. The active site residues are shown in pink-red sticks and Mg^2+^ ions are shown as green spheres

As the global COVID-19 pandemic has led to intense researchers on SARS-CoV-2, a number of groups have independently determined cryo-EM structures of the core RTC complexed with the RNA substrate and two nsp13 helicases, with nsp9 regulating the cap synthesis in addition, and also the core RTC bound with inhibitors, including the well-known remdesivir [58, 94–103]. In Fig. 4A, we show a composite structure of RTC (PDB accession codes: 7CXM, 6XEZ, 7CYQ), which includes nsp7, nsp8 (X2), nsp9, nsp12, nsp13 (X2), and RNA template and primer. In all RTC structures reported to date nsp12, nsp7, nsp8 and RNA primer and template duplex are identical, while nsp13 subunits have slight variations, and nsp9 is present in only one structure (PDB: 7CYQ). As the catalytical subunit of RTC, the RdRP domain of nsp12 (aa 325–932) binds the RNA duplex with the primer 3′ end docked in the active site formed by D618, D760 and D761. So far, all RdRP structures are devoid of an incoming NTP. Nsp12 contacts only 6 bp of RNA duplex upstream from the primer 3′ end (positions − 1 to − 6). Attached to the RdRP domain are two nsp8 subunits. Because the asymmetry nature of nsp12, nsp7 is needed to mediate the nsp8–nsp12 interactions on one side (Fig. 4A) [58, 94]. Nsp8 has a very long α-helix extended from the nsp8 globular domain interacting with nsp12 and nsp7 to the upstream RNA duplex. The pair of nsp8 helices are nearly parallel and hold the upstream RNA from positions − 10 to − 25 bp, thus stabilizing the core RTC–RNA interactions. Two nsp13 helicase molecules are loosely attached to the helical extensions of the two nsp8 above the RNA duplex (Fig. 4A). The active sites of nsp13 are marked by ADP·AlF_3_. The helicases have limited interactions with each other and appear to stabilize the overall architecture of RTC [98, 100]. One of the two nsp13 subunits is prone to dissociate in solution [98]. Nsp13_1_ helicase, which is attached to the nsp7/8 pair with additional interactions with the globular nsp8_1_ domain, also binds a disconnected downstream RNA template (5′ extension) at an orthogonal angle to the RNA duplex held by nsp12. If acting simultaneously, nsp13 and nsp12 would pull the RNA template in opposite ways (Fig. 4A) rather than in the same direction. It is unclear how the helicase may untangle structured RNA and feed it to RdRP for RNA synthesis.

Nsp12 also contains an N-terminal NiRAN (nidovirus RdRP-Associated Nucleotidyltransferase) domain (aa 1–250), which may transfer GMP to a 5′-ppA forming the 5′-GpppA cap. The nsp12 NiRAN domain is located distal from the RNA duplex, and a bound GDP marks its active site (Fig. 4A). It is suggested that nsp13 helicase removes the terminal phosphate from a 5′-pppA prior to GMP addition [104]. In the cryo-EM structure, nsp9 inserts its N- terminus into the NiRAN active site (Fig. 4A), which explains why nsp9 is NMPylation by NiRAN [56]. However, it is unclear how an RNA 5′-end displaces nsp9 for GMPylation.

The RdRP domain is a prime target for antiviral drugs. To date, several nucleotide analogs and non-nucleotide drugs have been found to inhibit the viral RNA replication and transcription. Remdesivir, the only FDA-approved drug for COVID-19 treatment [105], is a pro-drug containing a C1′-cyano substituted adenine and requires in vivo phosphorylation to form the active drug remdesivir triphosphate (RTP). After RTP is incorporated into a growing RNA product, it stalls RdRP because of steric clashes between the C1′-cyano group and Ser 861 (S861) (Fig. 4B) [95, 97, 103]. Another nucleotide analog Favipiravir mimics GTP and inhibits RTC by slowing down its own incorporation (Fig. 4C) [99]. Suramin is a non-nucleotide analog drug, and by having several SO_4_ groups it competes for the phosphate backbone-binding sites with both the template and primer (Fig. 4C) [101].

## Profiles of SARS-CoV-2 subgenomic RNAs in the infected cells

The template switch between TRS_L_ and TRS_B_ may be a good and simple model, which at least partially explains the SARS-CoV-2 RNA transcription and subgenome synthesis. This model also implies the template switching is inefficient, so the full-length gRNA is also transcribed. Because each viral RNA molecule is most likely in complex dynamically with RNA-binding proteins as an RNP (ribonucleoprotein complex) in the cytoplasm of infected cells, they are rarely naked at any given time during virus infection. Because TRS_L_ and TRS_B_ are very similar, some accessory factors and surrounding RNA sequence have to play a role to promote or suppress template switching. In fact, the nucleotide similarity between the TRS_B_ and TRS_L_ appears only partially important for a consequential interaction. Studies on Simian hemorrhagic fever virus, a close family member of *Coronaviridae*, have shown that not every TRS_B_ identified in the viral genome body is functional in the long-distance RNA–RNA interactions with the leader TRS_L_ to promote the template switch [106].

Varied transcription efficiency of individual sgRNAs is common in all coronaviruses. Recent RNA-seq analyses of SARS-CoV-2 infected Vero-E6 cells revealed the relative abundance of individual sgRNAs and junction sequence heterogeneity or “aberrant” template switches. The abundance of the individual SARS-CoV-2 sgRNAs identified by high quality TRS_L_–TRS_B_ junction reads both in the Vero-E6 and Caco-2 cells descended, interestingly, in the 3′ to 5′ direction of the viral genome, that is N, ORF8, ORF7a/b, M, ORF6, E, ORF3a, and S, with the N +sgRNA being the most and the S +sgRNA the least abundant [38, 78]. Also seen were TRS_B_-independent junctions of TRS_L_ and non-TRS dependent junctions in the infected cells [35, 38, 78] (Fig. 3B). It remains to be learnt whether RNA–RNA interactions independently of canonical TRS sequences along the SARS-CoV-2 genome inside cells could result in production of any sgRNAs and thereby diversify sgRNA populations.

**Fig. 5:**
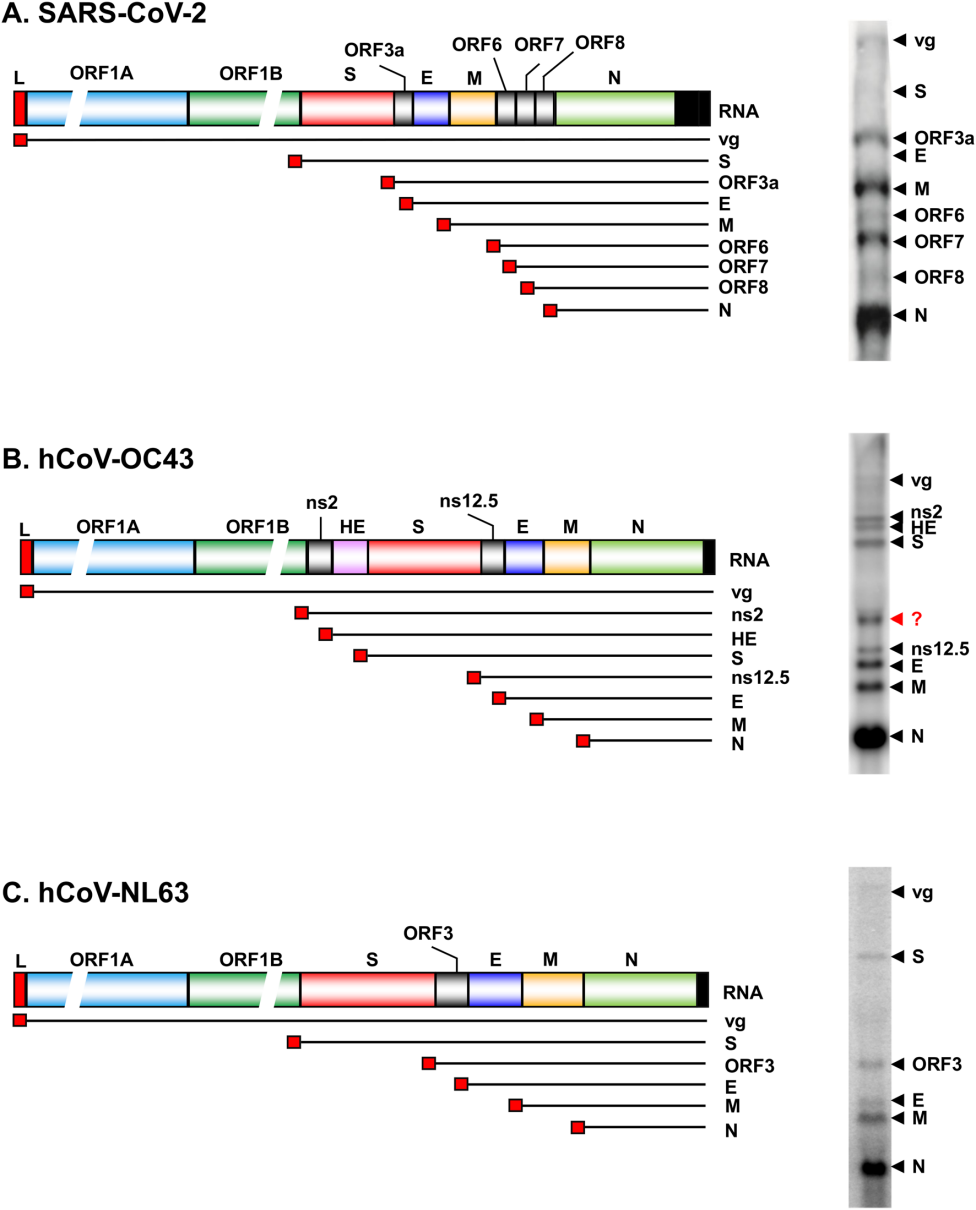
The expression of sgRNAs during human coronavirus infection. On the left are the diagrams of SARS-CoV-2 (**A**), hCoV-OC43 (**B**) and hCoV-NL63 (**C**) genomes and their coding potentials. Individual sgRNAs (lines) with a 5′ leader (small red box) obtained through the template switch are illustrated below and named by their corresponding proteins encoded. The viral gRNA (vgRNA) is generated by continuous transcription of the entire viral genome. On the right are the sgRNA expression profiles in African monkey kidney Vero E6 cells infected for 24 h with SARS-CoV-2 (**A**), 189 h with hCoV-NL63 (**C**), or human colorectal adenocarcinoma HCT-8 cells infected for 48 h with hCoV-OC43 (**B**). The sgRNAs detected by Northern blot analysis of total RNA extracted from infected cells using the individual antisense probes specific to each viral N gene. The Northern blot gel of SARS-CoV-2 sgRNA in **A** was modified with permission from a reference [38]

As detected by RNA-seq analyses, Northern blot analyses of SARS-CoV-2 infected cells using an antisense probes specific to the N gene region confirmed the production of most abundant viral N sgRNAs, followed by the sgRNAs of ORF7, ORF M and ORF3a [38] (Fig. 5A). Similarly, this approach in our studies of hCoV-OC43 and hCoV-NL63 infected cells also revealed the N sgRNAs being most abundant, followed by M and E sgRNAs (Fig. 5B, C), whereas the full-length viral gRNAs for virion assembly and the S sgRNAs for encoding viral spike protein were less abundant and sometimes barely detectable in the infected cells. A significant imbalance in abundance of the corresponding negative and positive sgRNAs was also observed [79]. The reason for this imbalanced production of sgRNA during virus infection is unclear and can’t be fully explained simply by poor base-pairing between TRS_L_–TRS_B_ interactions. The following hypothesis from our group offers a plausible interpretation: because RTC-initiated RNA transcription starts from the highly structured viral gRNA 3′ end, the first TRS_B_ encountered by RTC in transcribing RNA would be the TRS_B_ upstream of N gene. RTC pauses at the encountered terminal TRS_B_ in interacting with TRS_L_ and grasps the 5′ leader by template switch to produce the N sgRNAs. If leaky scanning or read-through occurs, the RTC continues scanning to further TRS_B_ upstream to define next sgRNA production by pausing and otherwise reads through the encountered TRS_B_. Since the TRS_B_ sequences toward the viral 5′ genome require more read-through steps to reach, it is conceivable that this scenario of “first come, first served” may explain why the N sgRNAs are the most abundant and the S sgRNA the less abundant. To transcribe a full-length gRNA, the RTC needs to read through all TRS_B_ sequences upstream of each ORF, thus resulting in less amount production of the full-length viral gRNA. It remains to know whether this hierarchical stoichiometry among individual sgRNAs is related to viral replication efficiency.

## Remarks and perspectives

The globally devastating COVID-19 pandemic by SARS-CoV-2 infection is an unprecedented public health disaster in human history in the modern time. After over a year of international efforts with more than 78,500 scientific publications by May 2, 2021 according to PubMed, remarkable progresses have been made in achieving the goals of preventing the pandemic by dispensing numerous SARS-CoV-2 vaccines to populations and treating the COVID-19 patients by antiviral compounds. The unprecedented mobilization of research funds and manpower in fighting the COVID-19 pandemic has resulted in rapidly growing knowledge about SARS-CoV-2 virus and its pathogenesis. Although the SARS-CoV-2 is no strange to us today, it remains to be known the virus origin and its intermediate animal hosts, and why it bursted out in the central China city Wuhan?

We have learned a great deal about each viral protein’s functions and structure by ectopic expression, but a chunk of basic knowledge on SARS-CoV-2 virology remains opaque. We know very little about this virus and its interactions with cellular machineries in host cells for its replication and transcription after virus infection. While this review focuses mainly on the progress in our understanding of SARS-CoV-2 genome structure, expression, and RTC mediated virus replication and transcription, we have also discussed many intriguing questions for future investigations in each section. The RNA template switch appears to be a simple, reasonable model to explain RTC-mediated production of sgRNAs during virus infection. However, to date, there is no direct experimental approach to verify the proposed transcriptional template switch.

Other remarkable questions also remain to be addressed. *Firstly*, all coronaviruses have a similar genome length and structure. However, high pathogenic SARS-CoV-2 and SARS-CoV encode more accessory proteins and thus produce more sgRNAs than the low pathogenic hCoV-OC43 and hCoV-NL63 in infected cells. Further studies are needed to understand if and how these additional accessory genes/sgRNAs contribute to pathogenesis and severity of SARS-related viral infections. *Secondly*, the full-length viral RNAs are only in a minimal amount compared to the abundant sgRNAs in the infected cells. However, only a single full-length +gRNA, but not sgRNAs, is needed for virion assembly. What is the driving force behind the specific selection of the full-length +gRNA from a mixed pool of +/−gRNAs and +/−sgRNAs, allowing a full-length +gRNA assemble into a virion? All +sgRNAs share the same 5′ leader and some parts of the 3′ RNA sequence with the full-length +gRNA, but no sgRNA could be enclosed into virions. We propose that the packaging signal (s) for successful virion assemble must exist within the region downstream of the 5′ leader, but upstream of the S ORF. *Thirdly*, although many cryo-EM structures of RTC have been determined, there are still many remaining questions regarding RTC structure and activity within the infected cells. For example, how nsp12 binds an incoming NTP and incorporates it into RNA; how nsp13 helicase facilitates RNA synthesis and cap formation; how the RNAs are capped by NiRAN; and whether other viral and host factors are involved in RTC formation and RNA synthesis is still unknown. To date, the multi-subunit RTC complex has been successfully drugged [99, 101, 105]. But all viral encoded proteins are potential targets for inhibition of SARS-CoV-2 infection. Inhibitors of proteases are currently in the pipeline [107–110]. We hope that inhibitors targeting necessary protein–protein interactions beyond viral enzymes will be developed as well.

SARS-CoV-2 infection and global COVID-19 scourge have taught us a painful and unforgettable lesson about how a tiny, invisible virus could rampage everyone’s daily life and paralyze our entire society in the modern world of the twenty-first century. With numerous, century-long discoveries and fundamental insights into biology of viruses and host cells they infect, virology has expanded the biomedical field in depth and breadth and laid the foundation of today’s molecular biology, structural biology, genome sciences, and precision medicine. These advances also led to prevention and even eradication of numerous life-threatening diseases. However, along with decoding the blueprint of human genome and emerging of various “seq” and imaging technologies and genome editing tools, many scientists and politicians thought that virology was a dying field and it was time to close the book on virology. After SARS-CoV in 2002, MERS-CoV in 2012 and SARS-CoV-2 in 2019, virus study is once again held in high reverence. We have finally come to realize that new viral pathogens will continue to emerge and we are living at a time of great need for the virology to understand the basic biology of viruses, virus–host interactions and harmony with nature and global ecosystem. The world needs to be prepared for emergence of possible SARS-CoV-3, SARS-CoV-4 or even other biological horrors because the question is not if but when they come [9, 111, 112].

## Data Availability

Not applicable.
